# Tuning Ag Loading and Particle Size in Ag@g-C_3_N_4_ Photocatalysts for Selective CO_2_ Conversion to CO and CH_4_

**DOI:** 10.3390/nano15181443

**Published:** 2025-09-19

**Authors:** Shicheng Liu, Na Li, Qulan Zhou

**Affiliations:** Multiphase Flow in Power Engineering, School of Energy and Power Engineering, Xi’an Jiaotong University, Xi’an 710049, China; scliu98@stu.xjtu.edu.cn (S.L.); lyna@mail.xjtu.edu.cn (N.L.)

**Keywords:** Ag nanoparticles, photocatalytic CO_2_ reduction, CO and CH_4_ pathway, DFT calculations, size-dependent selectivity

## Abstract

Elucidating the mechanisms of CO_2_ photocatalytic conversion systems is crucial for tackling the challenges of carbon neutrality. In this study, a series of Ag@g-C_3_N_4_ photocatalysts were constructed with metal particle size modulation as the core strategy to systematically reveal the modulation mechanism of Ag nanoparticles (Ag NPs) size variation on the selectivity of CO_2_ photoreduction products. Systematic characterizations revealed that increasing Ag size enhanced visible light absorption, promoted charge separation, and improved CH_4_ selectivity. Photocatalytic tests showed Ag_3.0%_@CN achieved optimal activity and electron utilization. Energy band analyses indicated that Ag modification preserved favorable conduction band positions while increasing donor capacity. Further density-functional theory (DFT) calculations reveal that Ag NPs size variations significantly affect the adsorption stability and conversion energy barriers of intermediates such as *COOH, CO and CHO, with small-sized Ag_7_ NPs favoring the CO pathway, while large-sized Ag NPs stabilize the key intermediates and drive the reaction towards the CH_4_ pathway evolution. The experimental and theoretical results corroborate each other and clarify the dominant role of Ag NPs size in regulating the reaction path between CO and CH_4_. This study provides mechanistic guidance for the selective regulation of the multi-electron reduction pathway, which is of great significance for the construction of efficient and highly selective CO_2_ photocatalytic systems.

## 1. Introduction

The escalating global carbon emissions crisis has made the achievement of carbon neutrality and establishment of sustainable low-carbon energy systems a paramount international priority in recent years [1,2]. CO_2_ photocatalytic reduction technology is considered to be one of the most promising paths for “artificial photosynthesis” as it can directly utilize solar energy to convert CO_2_ into high-value-added C_1_ chemicals (CO_2_ and CH_4_) to achieve greenhouse gas emission reductions and at the same time provide sustainable energy [3,4,5]. However, the multielectronic conversion process of CO_2_ (CO_2_ → CH_4_) is usually accompanied by complex intermediate migration and pathway bifurcation. The product distribution is strongly governed by critical catalyst structural parameters, including active component particle size, interfacial properties, and surface microenvironment characteristics [6,7]. Therefore, the precise modulation of catalyst structure, aiming at synergistically enhancing the photogenerated carrier separation efficiency and reactant activation process, has become a research hotspot. Further enhancement of the selective control of target products is one of the core challenges currently facing the field of photocatalytic CO_2_ reduction [8,9].

Graphite-phase carbon nitride (g-C_3_N_4_) is a non-metallic semiconductor with a medium bandgap (~2.7 eV), low synthesis cost and a broad visible-light response, which has been widely used in the photocatalytic reduction reaction of CO_2_ [10,11,12]. However, pristine g-C_3_N_4_ suffers from defects such as fast carrier complexation rate, poor reactive site activity, and weak interfacial electron mobility, which severely limit its photocatalytic performance [13,14]. Currently, researchers have widely used various modification studies such as vacancy engineering [15,16], Heteroatom doping [17,18], and construction of heterojunctions [19,20] to improve their defects. Among them, it was found that the photogenerated charge separation and surface reactivity were improved by introducing metal co-catalysts [21,22,23,24]. Benefiting from their LSPR-enhancing effect, high electron affinity and excellent CO_2_ adsorption capacity, silver nanoparticles (Ag NPs) show significant potential for applications in photocatalysis. Therefore, it is often employed in the g-C_3_N_4_ system to construct metal/semiconductor heterojunction structures to enhance CO_2_ photoreduction performance [25,26]. Chen et al. constructed plasmonic Ag NPs on nitrogen vacancy-modified g-C_3_N_4_ nanotubes (ACNNT) to achieve high photocatalytic conversion of CO_2_ under visible light. The uniform dispersion of Ag NPs coupled with the spatially oriented charge segregation and migration enabled by the one-dimensional tubular g-C_3_N_4_ architecture significantly enhances proton-assisted metal utilization efficiency [27].

Notably, the size variation in Ag NPs critically influences both their physicochemical stability and surface-active site distribution, while simultaneously governing the adsorption behavior and conversion pathways of key reaction intermediates (*CO, CHO). It has been pointed out that reducing the particle size of Ag NPs can enhance their specific surface area and metal-carrier interfacial interaction, thus improving the separation and transfer efficiency of photogenerated electrons. The above advantage makes them more prominent in driving the multi-electron hydrogenation reduction of CO_2_ and promoting the generation of deep reduction products such as CH_4_ [28]. Large-size Ag NPs, on the other hand, are more suitable for stabilizing key intermediates in the CO pathway because their electronic structure tends to be in the bulk state, thus enhancing the selective yield of CO [29]. However, current research has predominantly employed Ag NPs merely as auxiliary components. A fundamental knowledge gap persists regarding how particle size governs CO_2_ photoreduction pathway selectivity, particularly at the mechanistic level. This limited understanding substantially constrains the strategic application of metal particle size engineering in developing high-selectivity photocatalytic systems.

In this study, we constructed a series of g-C_3_N_4_ catalysts modified with Ag NPs of different particle sizes (Ag@g-C_3_N_4_) and systematically evaluated their effects on the activity and product distribution of the CO_2_ photo-reduction reaction under visible light. Controlled particle size growth of Ag NPs was achieved by modulating the loading of Ag. Combined with morphological characterization, photophysical property analysis, electronic structure determination and in situ reaction tests, the modulation of photogenerated electron separation efficiency, energy band structure evolution, and product path selection by Ag NPs size variation was systematically revealed. Complemented by density functional theory (DFT) calculations, we systematically compared the adsorption stability of key reaction intermediates and reaction energy barriers between small (Ag_7_) and large Ag NPs, revealing size-dependent catalytic effects. The linkage between the energy band structure evolution and the intermediate adsorption behaviors under the regulation of Ag NPs size has been further clarified to drive the change in product distribution. This study not only provides theoretical support for the understanding of the microscopic mechanism of Ag NPs size-regulated reaction pathways but also provides feasible strategies and mechanisms for the construction of highly selective CO_2_ photoreduction systems.

## 2. Materials and Methods

### 2.1. Catalyst Preparation

g-C_3_N_4_ was prepared by thermal polymerization. Weighing 10 g of urea (Aladdin, 99%), placed in an alumina crucible with a lid, heated to 550 °C in a muffle furnace at a heating rate of 5 °C/min and held for 2 h. After cooling, a light yellow mass was obtained, and g-C_3_N_4_ powder was obtained by grinding and sieving [30]. Ag@g-C_3_N_4_ catalyst was prepared by chemical reduction method. 100 mg of g-C_3_N_4_ was dispersed in 100 mL of deionized water and sonicated for 30 min to obtain a homogeneous dispersion. Subsequently, different proportions of AgNO_3_ solution (corresponding to Ag loading of 0.5%, 1.0%, 3.0%, and 5.0%) were added, and an appropriate amount of NaBH_4_ solution was added dropwise as a reducing agent after stirring well. After stirring and reacting the system in the dark for 2 h, Ag@g-C_3_N_4_ solid powder was obtained by centrifugation, washing and drying.

### 2.2. Structural Characterization of Catalysts

The samples were characterized using transmission electron microscopy (TEM; JEM-2100, JEOL Ltd., Tokyo, Japan) and high-resolution TEM (HRTEM) to examine morphology and microstructure. Crystalline features were analyzed through selected-area electron diffraction (SAED) with corresponding fast Fourier transform (FFT) patterns. Elemental distribution mapping was performed using scanning transmission electron microscopy with energy-dispersive X-ray spectroscopy (STEM-EDS). X-ray diffraction (XRD) patterns were collected on a Bruker D8 Advance diffractometer with Cu Kα radiation (λ = 1.5406 Å). Surface area measurements were obtained from N_2_ adsorption–desorption isotherms (Micromeritics ASAP 2020) using the BET method. Optical properties were evaluated by ultraviolet-visible diffuse reflectance spectroscopy (UV–Vis DRS; UV-2600, Shimadzu Corporation, Kyoto, Japan), while photoluminescence (PL) spectra were recorded on a Horiba Fluorolog-3 spectrofluorometer (excitation at 325 nm). Chemical states were analyzed by X-ray photoelectron spectroscopy (XPS; ESCALAB 250Xi, Thermo Fisher Scientific, Waltham, MA, USA), with valence band (VB) spectra providing energy band structure information.

### 2.3. Photocatalytic Performance Test

The CO_2_ photoreduction reaction was carried out in a closed quartz reaction system consisting of 100 mg of catalyst, 20 mL of deionized water and 100 mL of CO_2_, with the reaction gas pre-filled with the quartz reactor. The light source was a 300 W xenon lamp (Xenon lamp; PLS-SXE300, Perfectlight Technology Co., Ltd., Beijing, China) with an irradiation wavelength range of 320–780 nm and a light intensity of 100 mW-cm^−2^, simulating solar lighting conditions. The reaction temperature was maintained at 25 °C. Reaction products were sampled hourly and analyzed by gas chromatography (FULI-F80, equipped with FID and TCD detectors) to quantify CO and CH_4_ yields. All experimental results represent the average of three independent measurements.

### 2.4. Density Functional Theory Calculations

Density Functional Theory (DFT) calculations are accomplished in the Vienna abinitio simulation package (VASP2.4.6) software, using the projective affixed plus wave (PAW) method for the pseudopotentials, while the exchange-correlation generalization takes the form of the PBE in the Generalized Gradient Approximation (GGA) [31]. The plane-wave cutoff energy was set to 450 eV, with Γ-point sampling of the system momentum space. A convergence threshold of 10^−5^ eV was employed for all calculations. The simulation models include the structures of pure g-C_3_N_4_ sheet layers, loaded Ag_7_ NPs and Ag large-size NPs, and all models are constructed at vacuum layer thicknesses greater than 15 Å [32,33]. The adsorption and reaction free energies of each key intermediate in the CO_2_ reduction pathway (*COOH, *CO, *CHO, *CH_4_) were evaluated by calculating ΔG, with the temperature set to 298 K, using the standard free energy correction method.

## 3. Results and Discussion

### 3.1. Structural Characteristics

**Figure 1:**
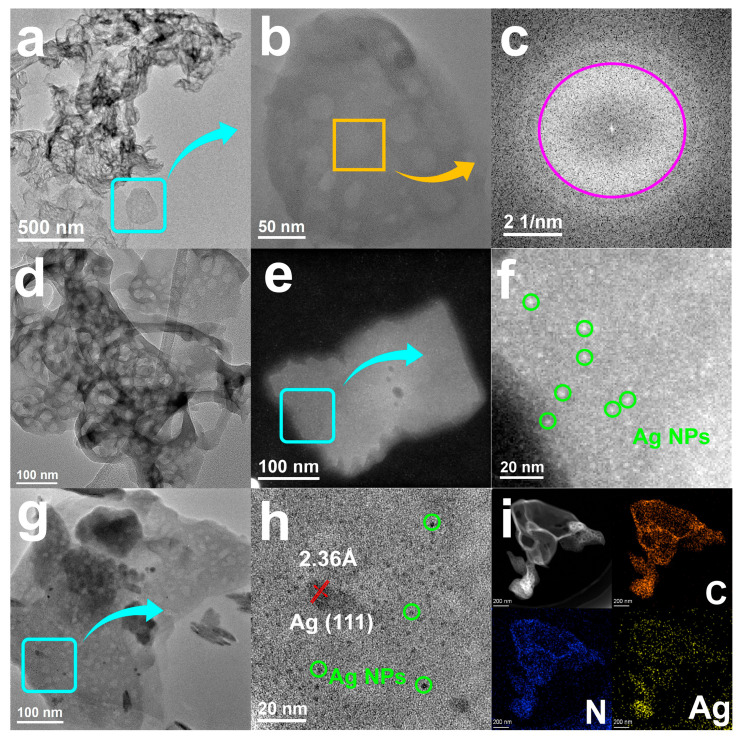
(**a**) HAADF-STEM image and (**b**) Magnified view of g-C_3_N_4_. (**c**) FFT images of g-C_3_N_4_. (**d**) HAADF-STEM image of Ag_0.5%_@CN. (**e**) HAADF-STEM image and (**f**) Magnified view of Ag_1.0%_@CN. (**g**) HAADF-STEM image and (**h**) Magnified view of Ag_3.0%_@CN. (**i**) Elemental maps of Ag_5.0%_@CN.

Figure 1a demonstrates the overall morphology of the pure g-C_3_N_4_ sample, which is structured as a typical lamellar body with regular edges, and no obvious particles attached to the surface. Further observation of the local magnification as shown in Figure 1b reveals that the thickness of the lamellae is uniform and the boundaries are well defined. Figure 1c shows the fast Fourier transform (FFT) image of the corresponding region of Figure 1b, which shows only fuzzy amorphous diffraction rings and no clear lattice spots, indicating that g-C_3_N_4_ as a whole is amorphous and lacks long-range ordered structure, providing a baseline for the subsequent observation of the crystalline characteristics of Ag NPs [34]. On this basis, the morphological evolution of the samples with different silver loadings was further investigated. As shown in Figure 1d, the Ag_0.5%_@CN sample maintains the characteristic lamellar morphology of g-C_3_N_4_ with smooth surfaces. No distinct Ag NPs were resolved within the observed field of view, likely due to either their ultrasmall size or low electron density contrast. As the silver content rises to 1.0%, a number of low-contrast dark spots appear in Figure 1e, indicating that Ag NPs are beginning to form, although the morphology is still blurred. Figure 1f presents a high-magnification view of the region marked in Figure 1e, clearly revealing well-dispersed Ag NPs with diameters below 5 nm on the g-C_3_N_4_ surface. The NPs exhibit well-defined crystallinity and uniform dispersion, confirming the successful incorporation of small-sized silver particles with excellent spatial distribution.

Continuing to increase the percentage of silver loading, the distribution density and size of Ag NPs in the samples changed significantly. As shown in Figure 1g, a large number of contrast-enhanced particles were attached to the surface of the lamellae in the Ag_3.0%_@CN samples, with a significant increase in the number. At the same time, the particle spacing was significantly reduced, reflecting higher nucleation density. Figure 1h further magnified observation reveals the presence of several large-sized particles in addition to the small-sized Ag NPs, with lattice stripes clearly visible within the particles. The measured crystal plane spacing is about 2.36 Å, which corresponds to the (111) crystal plane of metallic silver, indicating that some of the particles have grown into highly crystalline nanocrystals [35]. Figure 1i displays the EDS elemental mapping of the Ag_5.0%_@CN sample, showing uniform distribution of C and N across the lamellar framework. The Ag signals exhibit continuous spatial dispersion without apparent aggregation or vacancy regions, demonstrating that structural integrity and nanoparticle dispersion are effectively preserved even at high loading concentrations.

**Figure 2:**
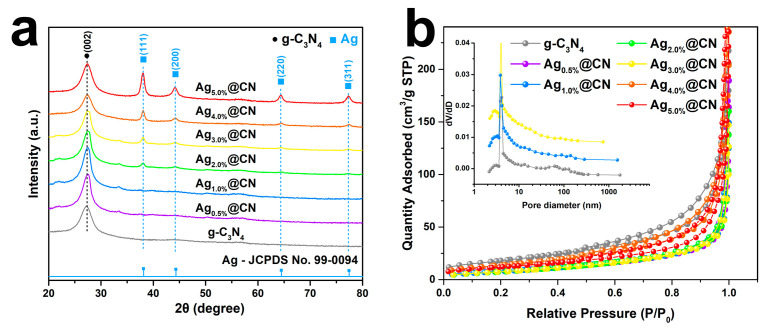
(**a**) XRD patterns and (**b**) N_2_ adsorption–desorption isotherms of g-C_3_N_4_ and Ag-loaded g-C_3_N_4_ samples.

The XRD patterns of g-C_3_N_4_ and Ag@g-C_3_N_4_ with varying Ag loadings are presented for comparison (Figure 2a). Pure g-C_3_N_4_ exhibits a distinct diffraction peak at 2θ = 27.5°, corresponding to the (002) crystal plane, which reflects its stacking structure along the interlayer direction [36]. Following Ag incorporation, while retaining the dominant g-C_3_N_4_ diffraction features, new peaks gradually emerged at 38.1°, 44.3°, 64.4°, and 77.4°. These peaks correspond to the (111), (200), (220), and (311) crystallographic planes of metallic Ag (JCPDS No. 99-0094), respectively [37]. The Ag diffraction peak intensities exhibited a loading-dependent enhancement, becoming particularly prominent in Ag_3.0%_@CN and higher-loading samples. The dominant Ag (111) peak evolution indicates both improved crystallinity and particle growth beyond the XRD detection threshold. In addition, no other impurity phases or relevant features of the oxidized state of silver were seen, suggesting that the introduced Ag is mainly present in the system as a zero-valent metal.

As shown in Figure 2b, all samples showed typical type IV adsorption isotherms, with a significant increase in the P/P0 ≈ 0.9–1.0 interval, indicating that the samples have a mesoporous structure [38]. Pristine g-C_3_N_4_ exhibits the highest specific surface area (65.6 m^2^ g^−1^) and pore volume (0.222 cm^3^ g^−1^), indicative of a well-developed porous structure. Upon the gradual introduction of Ag, the adsorption isotherms shift downward, and the overall adsorption capacity decreases markedly, suggesting that Ag NPs partially occupy or block the pores of g-C_3_N_4_, thereby reducing the accessible surface area. The pore size distribution further supports this observation. Pristine g-C_3_N_4_ exhibits a clear mesoporous feature centered at ~13 nm, while Ag@CN samples show weakened peaks accompanied by a slight shift toward smaller pore sizes. As summarized in Table 1, further increasing the Ag loading to 3.0–5.0wt% leads to a modest recovery of surface area and pore volume, with the pore size approaching its original value. This trend indicates that at higher Ag loadings, larger Ag particles tend to grow on the outer surface rather than infiltrating the pores, thereby alleviating the blocking effect and allowing the intrinsic mesoporous framework of g-C_3_N_4_ to be partially preserved. This trend indicates that at higher Ag loadings, larger Ag NPs preferentially grow on the outer surface rather than infiltrating the pores. As a result, the blocking effect is alleviated, and the intrinsic mesoporous framework of g-C_3_N_4_ is partially preserved. These structural changes are consistent with the TEM observations, which reveal particle agglomeration and diminished dispersion at high loadings. While elevated Ag loading enhances reactive site density, it concurrently induces structural pore occlusion and light shielding effects.

### 3.2. Photocatalytic Performance Evaluation

**Figure 3:**
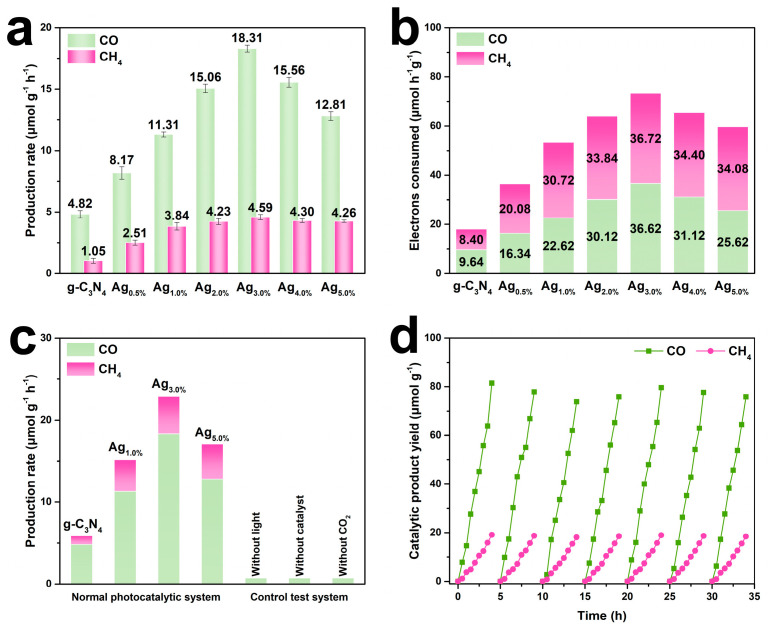
(**a**) CO and CH_4_ production rates and (**b**) electron consumption amounts of different samples. (**c**) Comparative tests under different control conditions (without light, catalyst, or CO_2_). (**d**) Photocatalytic stability of Ag_3.0%_@CN over 7 consecutive cycles. (Reaction conditions: 100 mg catalyst, 20 mL deionized water, and 100 mL CO_2_ in a closed quartz reactor; light source: 300 W Xe lamp (320–780 nm, 100 mW cm^−2^); reaction temperature: 25 °C; products analyzed hourly by gas chromatography).

As shown in Figure 3a, the catalysts with different Ag loadings exhibited significant differences in CO and CH_4_ yields. Pure g-C_3_N_4_ achieved only 4.82 μmol-g^−1^-h^−1^ CO and 1.05 μmol-g^−1^-h^−1^ CH_4_ yields, indicating that its limited intrinsic activity. The catalytic activity increased significantly with the introduction of Ag NPs. The Ag_3.0%_@CN sample showed the best performance, with CO and CH_4_ production rates of 18.31 and 4.59 μmol-g^−1^-h^−1^, respectively. Continuing to increase the Ag loading to 4.0% versus 5.0%, the gas production performance instead decreased, suggesting that a moderate amount of Ag could promote CO_2_ reduction, whereas excessive loading could cause carrier complexation or masking of light absorption. The electron consumption in the catalytic reaction of each sample also showed a similar trend as shown in Figure 3b. The electron consumption was determined based on the quantified CO and CH_4_ yields, with 2 electrons consumed per CO molecule and 8 electrons consumed per CH_4_ molecule. Notably, Ag_3.0%_@CN consumed the highest total electrons (73.34 μmol-h^−1^-g^−1^). The proportion of electrons involved in the CH_4_ pathway also increased significantly, suggesting that the electron utilization efficiency and reduction depth were both optimal at this loading.

In order to verify the real contribution of Ag loading to the photoreduction performance of CO_2_, the gas production behaviors under different systems are compared in Figure 3c. Under standard conditions, the Ag_1.0%_, Ag_3.0%_ and Ag_5.0%_@CN samples all exhibited higher catalytic ability than the g-C_3_N_4_ native, and Ag_3.0%_@CN again demonstrated the best performance. In the control experiments, gas production was almost zero if light, catalyst, or CO_2_ was removed, indicating that the reaction was indeed a light-driven CO_2_ reduction promoted by Ag NPs-modified g-C_3_N_4_ catalyst. Figure 3d further examines the stability behavior of the Ag_3.0%_@CN sample under cyclic reaction conditions. As the reaction time was extended, the CO and CH_4_ yields showed good reproducibility in seven consecutive rounds of reaction, and the cumulative production increased basically linearly. No obvious activity decay was observed during the reaction, suggesting that the catalyst has excellent structural stability and catalytic durability under long-time light conditions.

### 3.3. Electronic Structure and Band Modulation

**Figure 4:**
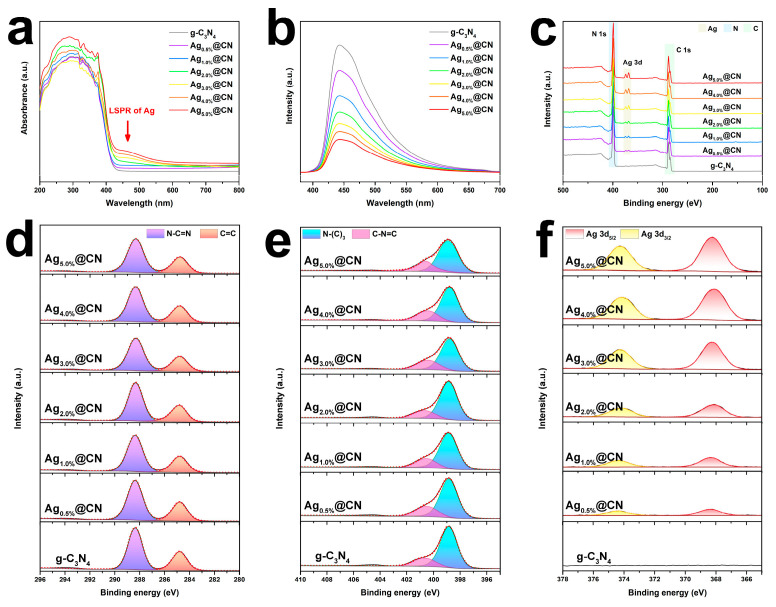
(**a**) UV–Vis diffuse reflectance spectra of g-C_3_N_4_ and Ag_x%_@CN (**b**) PL spectra of g-C_3_N_4_ and Ag_x%_@CN. (**c**) XPS survey spectra of all samples. High-resolution (**d**) C 1s and (**e**) N 1s spectra of all samples. (**f**) Ag 3d spectra of all samples.

The UV-visible diffuse reflectance spectra (Figure 4a) reveal strong visible-light absorption across all samples, demonstrating excellent photoresponsive characteristics. Ag incorporation further enhanced absorption intensity, likely due to the LSPR effects. Pure g-C_3_N_4_ shows a pronounced absorption edge near 400 nm, attributed to its intrinsic π-π* electron jump, corresponding to a band gap of about 2.7 eV [30]. As the Ag loading increased, the catalyst exhibited no appreciable absorption edge redshift but demonstrated progressive enhancement of broadband absorption between 450 and 500 nm, which was most prominent in Ag_3.0%_@CN and Ag_5.0%_@CN. This phenomenon can be attributed to the LSPR effect of Ag NPs, which induces new absorption bands that help to enhance the light harvesting capability and extend the light response range for subsequent photogenerated electron generation and transfer.

As shown in Figure 4b, all the samples show obvious broad emission peaks around 470 nm, originating from the radiative complexation process of photogenerated electrons and holes [39]. Of them, the g-C_3_N_4_ body has the highest PL intensity, suggesting a serious carrier complexation problem. Whereas, after Ag loading, the PL intensity of the samples decreases gradually with the increase in Ag NPs. This reflects that the non-radiative composite process is enhanced and electrons are more likely to migrate towards Ag, which inhibits the composite behavior. Among all samples, Ag_5.0%_@CN exhibits the lowest PL intensity. However, its excessive Ag loading induces NPs agglomeration and partial surface coverage, which block active sites and hinder efficient charge utilization. Notably, Ag_3.0%_@CN exhibits the highest photocatalytic activity, indicating an optimal balance between suppressed carrier recombination and plentiful accessible active sites.

To further confirm the Ag introduction and its valence information, the full XPS survey spectrum of each sample is given in Figure 4c. All samples exhibit distinct C 1s (≈284 eV) and N 1s (≈399 eV) major peaks, constituting the basic skeleton of g-C_3_N_4_ [40]. For the Ag-modified samples, Ag 3d double peaks were observed in the region of 368–374 eV, and their peak intensities were enhanced with the increase in loading. This reflects a controlled increase in Ag NPs, showing that Ag was successfully introduced and existed in a surface stable state [30].

Figure 4d illustrates the high-resolution spectrum of C 1s, which mainly contains two fitted components at 288.3 eV (N-C=N) and 284.8 eV (C-C/C=C), representing the presence of C atoms in the triazine ring with a small amount of carbon-rich regions, respectively [41,42]. The position of the main peak was not significantly shifted in each sample, indicating that the introduction of Ag did not disrupt the carbon skeleton structure of g-C_3_N_4_.

Figure 4e shows a high-resolution XPS image of N 1s, which can be divided into two peaks: 398.6 eV corresponds to sp^2^ hybridized nitrogen (N-(C)_3_), and 400.1 eV corresponds to bridged nitrogen (C-N-C) [27]. The stable peak positions and similar intensities between the different Ag-loaded samples illustrate that the Ag deposition process did not significantly change the chemical environment of elemental nitrogen, with the structural integrity of g-C_3_N_4_ being maintained.

To elucidate the chemical valence states of Ag, the corresponding results are displayed in Figure 4f. In all Ag-modified samples, Ag 3d_5/2_ and Ag 3d_3/2_ are located at 368.2 eV and 374.2 eV, respectively, with a spacing of about 6.0 eV, which is in accordance with the characteristics of the metallic state Ag^0^ [43]. No additional peaks representing Ag^+^ or AgO_x_ in the 373–378 eV region were observed, suggesting that Ag exists predominantly as an unoxidized metal. Combining the LSPR absorption features with the PL bursting behavior in Figure 4a,b, the Ag NPs not only exist stably on the surface, but also act as electron acceptors and participate effectively in the electron transfer process.

**Figure 5:**
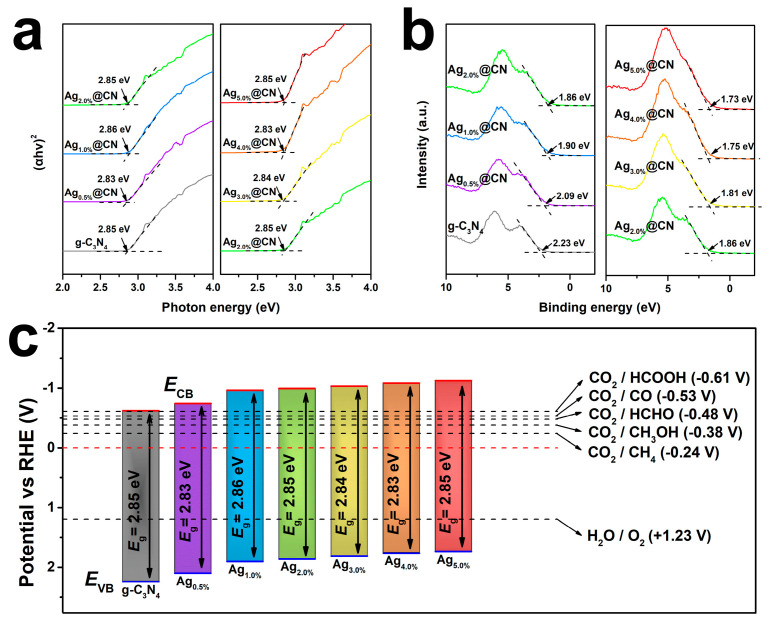
(**a**) Tauc’s plots for band gap estimation of g-C_3_N_4_ and Ag_x%_@CN. (**b**) Valence band of g-C_3_N_4_ and Ag_x%_@CN. (**c**) Schematic energy band diagrams of g-C_3_N_4_ and Ag_x%_@CN.

The Tauc method was applied to fit the optical bandgaps of g-C_3_N_4_ and the Ag-loaded samples (Figure 5a). The band gap of the pure g-C_3_N_4_ sample is 2.85 eV, which exhibits typical wide bandgap semiconductor characteristics. Upon Ag incorporation, the samples exhibit no significant absorption edge redshift, maintaining bandgap values between 2.83 and 2.86 eV. This confirms that Ag loading preserves the fundamental π-conjugated framework of g-C_3_N_4_ while introducing additional light-harvesting capabilities [44]. The slight narrowing of Ag_0.5%_@CN to 2.83 eV is related to its formation of surface-isolated excitonic coupling states. Overall, the Ag modification did not break the main framework of light absorption of g-C_3_N_4_ and ensured the catalyst’s light-absorbing ability in the visible region.

The XPS valence band spectrum of each sample is shown in Figure 5b, showing the position of its valence band top (VB) relative to the Fermi energy level. The g-C_3_N_4_ body has the largest VB of about 2.23 eV, and the position of the valence band is gradually shifted towards lower energies with increasing Ag NPs [45,46]. With the gradual increase in Ag loading from 0.5% to 5.0%, the valence band positions of the samples decreased to 2.09, 1.90, 1.86, 1.81, 1.75 and 1.73 eV in sequence, suggesting that the introduction of Ag effectively modulates the surface energy level structure of the materials while enhancing the electron density. The lower VB position facilitates the enhancement of the migration potential energy of the electrons on the reducing side, which enhances the CO_2_ molecule activation and its intermediate conversion.

The energy band structure evolution of the Ag@g-C_3_N_4_ sample is constructed in Figure 5c based on the data of Figure 5a with Figure 5b and superimposed on the standard potentials of a typical CO_2_ reduction half-reaction [47,48]. As seen in the figure, the conduction band bottoms (CB) of all the samples are located above the potentials required for the reduction of CO_2_ to produce CH_4_ (−0.24 V), CH_3_OH (−0.38 V), and CO (−0.53 V), showing that they have thermodynamic driving capability. In particular, the energy band structure of Ag_3.0%_@CN has both a relatively suitable band gap and a high conduction band potential, which combines light absorption and electron reduction, matching the performance of the optimal yield in the aforementioned catalytic experiments. Furthermore, the elevated valence band position exceeds the H_2_O/O_2_ oxidation potential (+1.23 V), guaranteeing thermodynamic favorability for the oxidative half-reaction. This optimized band alignment provides comprehensive energetic support for the multi-electron CO_2_ reduction pathway.

### 3.4. DFT Calculations

**Figure 6:**
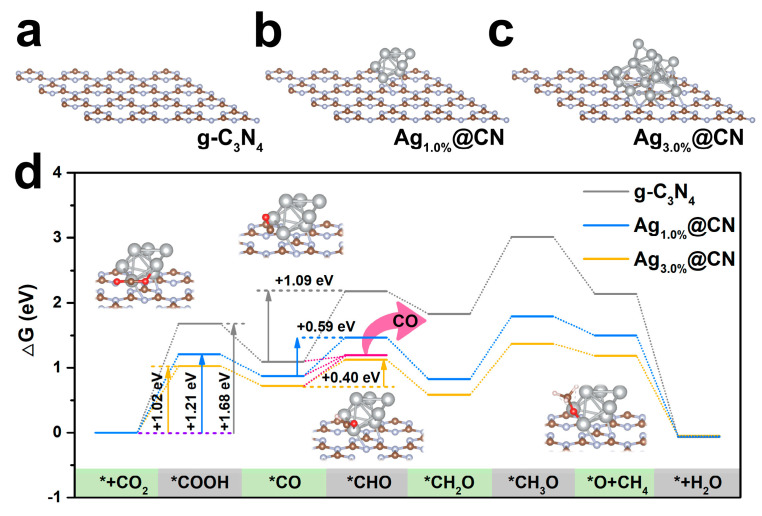
Optimized structure of (**a**) g-C_3_N_4_, (**b**) Ag_1.0%_@CN and (**c**) Ag_3.0%_@CN. (**d**) Free energy profiles of g-C_3_N_4_, Ag_1.0%_@CN and Ag_3.0%_@CN.

To elucidate the effect of Ag loading, the g-C_3_N_4_, Ag_1.0%_@CN and Ag_3.0%_@CN samples are considered as theoretical model structures, enabling a comparison of the modulation of reactive center and electronic structure. Figure 6a shows a model of the native g-C_3_N_4_ with a regular lamellar structure and exposed N sites on the surface, constituting the basic adsorption and reaction interface. The computational model of Ag_7_ NPs anchored on g-C_3_N_4_, representing the Ag_1.0%_@CN system with characteristic smaller Ag NPs, is shown in Figure 6b. The NPs adopts a bridging configuration between two adjacent nitrogen sites, forming a stable interfacial architecture while maintaining accessible metal active centers. Figure 6c displays the Ag_18_ NPs model representing the Ag_3.0%_@CN system, where increased NP dimensions create additional low-coordination sites. This structural configuration is anticipated to significantly strengthen CO_2_ adsorption activation and subsequent intermediate stabilization during reduction.

Figure 6d illustrates the Gibbs free energy (ΔG) change curves of each intermediate during CO_2_ photoreduction for the three model systems, which covers a multi-step reduction path from CO_2_ adsorption to CH_4_ desorption. In the g-C_3_N_4_ model, COOH formation is the main rate-limiting step (ΔG = 1.68 eV), and the CO state adsorption is less stable and easy to desorb, so that it prefers to generate CO products [49]. After the introduction of Ag_7_ NPs into the Ag_1.0%_@CN model, the free energy curves shifted downward overall, especially on the formation of two key intermediates, COOH and CO, ΔG was significantly reduced, with the former dropping to 1.21 eV and the latter stabilized below 0.59 eV. This suggests that small-sized Ag NPs play a positive role in lowering the electron transfer barrier and enhancing the preliminary reduction activity [50,51]. DFT calculations reveal that Ag_1.0%_@CN lowers the energy barrier for CO_2_ photoreduction, thereby contributing to its overall enhanced photocatalytic activity compared with g-C_3_N_4_. After further increasing the Ag loading to form larger NPs, the Ag_3.0%_@CN system showed a clear advantage in the reaction path. The ΔG for initial COOH formation decreases significantly to 1.02 eV, while subsequent reaction barriers along the entire CO→CH_4_ pathway show systematic reduction.

Additionally, Ag_3.0%_@CN shows a higher CO desorption free energy (+0.47 eV) than Ag_1.0%_@CN (+0.32 eV), making CO desorption less favorable and thereby facilitating subsequent hydrogenation. Although ΔG (CO desorption) is higher than ΔG (*CHO formation) in Ag_3.0%_@CN, experimental results show that CO remains the primary product of CO_2_ photoreduction. Direct *CO desorption represents a shorter pathway, rapidly generating CO. In contrast, although hydrogenation to *CHO is thermodynamically accessible, it requires additional steps to ultimately form CH_4_, making this process less favorable. As a result, CO remains the main photocatalytic product. In summary, the DFT free energy analysis in Figure 6d clearly reveals the regulatory mechanism of Ag NPs size on reaction path selection. Smaller Ag NPs primarily lower the initial reduction barrier. In contrast, larger Ag NPs (Ag_3.0%_@CN) create a more electron-rich interface and strengthen CO adsorption on the catalyst surface, directing the reaction toward the deep reduction pathway leading to CH_4_. This size-dependent conformational relationship provides theoretical support for the experimentally observed trends in the distribution of CO and CH_4_.

## 4. Conclusions

This work systematically demonstrates that Ag nanoparticle size plays a key role in modulating the photocatalytic CO_2_ reduction behavior of g-C_3_N_4_. Regulating the Ag loading allows control over the size of Ag NPs on g-C_3_N_4_, which in turn governs the photocatalytic activity. Smaller Ag NPs (Ag_1.0%_@CN) facilitate CO desorption due to weaker intermediate adsorption, favoring CO production. In contrast, larger Ag NPs (Ag_3.0%_@CN) stabilize CO intermediates and lower the hydrogenation barrier, promoting CH_4_ formation. Spectroscopic characterizations confirm that Ag enhances visible light absorption via LSPR and improves charge separation efficiency without altering the intrinsic bandgap of g-C_3_N_4_. Combined XPS and UV–Vis analyses reveal that Ag modification modulates surface energy levels and improves electron reduction potential, meeting the thermodynamic requirements for multi-electron transfer. DFT calculations further show that small Ag_7_ NPs reduce CO desorption energy, while large Ag NPs create electron-rich interfaces that stabilize key intermediates and facilitate CH_4_ evolution. These results establish a structure–function relationship linking Ag size with energy band structure, intermediate binding strength, and product pathway differentiation. The study provides mechanistic insights and structural guidance for designing highly selective CO_2_ photocatalysts based on g-C_3_N_4_ platforms.

## Figures and Tables

**Table 1:** Surface area (SBET), Pore Volume (Vpore) and Pore Diameter (Dpore) of pristine g-C3N4 and Ag-loaded g-C3N4 samples.

Catalyst	S_BET_ (m^2^ g^−1^)	V_pore_ (cm^3^ g^−1^)	D_pore_ (nm)
g-C_3_N_4_	65.629	0.222	13.532
Ag_0.5%_@CN	53.394	0.177	12.450
Ag_1.0%_@CN	43.476	0.139	11.596
Ag_2.0%_@CN	47.153	0.156	12.359
Ag_3.0%_@CN	49.746	0.185	12.526
Ag_4.0%_@CN	50.751	0.191	13.311
Ag_5.0%_@CN	52.744	0.204	13.543

## Data Availability

The data that support the findings of this study are available from the corresponding author upon reasonable request.

## References

[1] Acharya R., Parida K. (2020). A review on TiO_2_/g-C_3_N_4_ visible-light- responsive photocatalysts for sustainable energy generation and environmental remediation. J. Environ. Chem. Eng..

[2] Ortiz-Quiñonez J.-L., Pal U. (2024). Interface engineered metal oxide heterojunction nanostructures in photocatalytic CO_2_ reduction: Progress and prospects. Coord. Chem. Rev..

[3] Takata T., Jiang J., Sakata Y., Nakabayashi M., Shibata N., Nandal V., Seki K., Hisatomi T., Domen K. (2020). Photocatalytic water splitting with a quantum efficiency of almost unity. Nature.

[4] Liu X., Inagaki S., Gong J. (2016). Heterogeneous Molecular Systems for Photocatalytic CO_2_ Reduction with Water Oxidation. Angew. Chem. Int. Ed..

[5] Fang S., Rahaman M., Bharti J., Reisner E., Robert M., Ozin G.A., Hu Y.H. (2023). Photocatalytic CO_2_ reduction. Nat. Rev. Methods Primers.

[6] Guo Z., Chen G., Cometto C., Ma B., Zhao H., Groizard T., Chen L., Fan H., Man W.-L., Yiu S.-M. (2019). Selectivity control of CO versus HCOO− production in the visible-light-driven catalytic reduction of CO_2_ with two cooperative metal sites. Nat. Catal..

[7] Rao H., Schmidt L.C., Bonin J., Robert M. (2017). Visible-light-driven methane formation from CO_2_ with a molecular iron catalyst. Nature.

[8] Xu S., Li X., Li S., Rao H., Qin J.-s., She P., Cheong W.-C., Jing L. (2025). Recent Advances of Photocatalytic CO_2_ Reduction Based on Hybrid Molecular Catalyst/Semiconductor Photocatalysts: A Review. Small.

[9] Luo Y., Zhu Y., Han Y., Ye H., Liu R., Lan Y., Xue M., Xie X., Yu S., Zhang L. (2023). g-C_3_N_4_-based photocatalysts for organic pollutant removal: A critical review. Carbon Res..

[10] Mao L., Zhai B., Wen L., Xiao W., Shi J., Kang X., Liu Y., Cheng C., Jin H., Guo L. (2025). Simultaneous bulk and surface modifications of g-C_3_N_4_ via supercritical CO_2_ -assisted post-treatment towards enhanced photocatalytic activity. Appl. Catal. B Environ. Energy.

[11] Yu W., You X., Wu Y., Li C., Wang Q., Yang X., Bian X., Lu Y. (2025). Achieving highly efficient and selective CO_2_ photoreduction to CO over g-C_3_N_4_ via micro-environment optimization mediated by HZSM-5 zeolite integration. J. Environ. Chem. Eng..

[12] Wang Q., Wang X., Yu Z., Jiang X., Chen J., Tao L., Wang M., Shen Y. (2019). Artificial photosynthesis of ethanol using type-II g-C_3_N_4_/ZnTe heterojunction in photoelectrochemical CO_2_ reduction system. Nano Energy.

[13] Ye L., Wu D., Chu K.H., Wang B., Xie H., Yip H.Y., Wong P.K. (2016). Phosphorylation of g-C_3_N_4_ for enhanced photocatalytic CO_2_ reduction. Chem. Eng. J..

[14] Tang J., Guo C., Wang T., Cheng X., Huo L., Zhang X., Huang C., Major Z., Xu Y. (2024). A review of g-C_3_N_4_-based photocatalytic materials for photocatalytic CO_2_ reduction. Carbon Neutralization.

[15] Liu Y., Zhao L., Zeng X., Xiao F., Fang W., Du X., He X., Wang D., Li W., Chen H. (2023). Efficient photocatalytic reduction of CO_2_ by improving adsorption activation and carrier utilization rate through N-vacancy g-C_3_N_4_ hollow microtubule. Mater. Today Energy.

[16] Song Q., Hu J., Zhou Y., Ye Q., Shi X., Li D., Jiang D. (2022). Carbon vacancy-mediated exciton dissociation in Ti3C2Tx/g-C_3_N_4_ Schottky junctions for efficient photoreduction of CO_2_. J. Colloid Interface Sci..

[17] Li Q., Wang S., Sun Z., Tang Q., Liu Y., Wang L., Wang H., Wu Z. (2019). Enhanced CH4 selectivity in CO_2_ photocatalytic reduction over carbon quantum dots decorated and oxygen doping g-C_3_N_4_. Nano Res..

[18] Park J., Liu H., Piao G., Kang U., Jeong H.W., Janáky C., Park H. (2022). Synergistic conversion of CO_2_ into C1 and C2 gases using hybrid in-doped TiO_2_ and g-C_3_N_4_ photocatalysts. Chem. Eng. J..

[19] Que M., Cai W., Chen J., Zhu L., Yang Y. (2021). Recent advances in g-C_3_N_4_ composites within four types of heterojunctions for photocatalytic CO_2_ reduction. Nanoscale.

[20] Wang J., Yu Y., Cui J., Li X., Zhang Y., Wang C., Yu X., Ye J. (2022). Defective g-C_3_N_4_/covalent organic framework van der Waals heterojunction toward highly efficient S-scheme CO_2_ photoreduction. Appl. Catal. B Environ..

[21] Sun J., Bian J., Li J., Zhang Z., Li Z., Qu Y., Bai L., Yang Z.-D., Jing L. (2020). Efficiently photocatalytic conversion of CO_2_ on ultrathin metal phthalocyanine/g-C_3_N_4_ heterojunctions by promoting charge transfer and CO_2_ activation. Appl. Catal. B Environ..

[22] Liu G., Wang Y., Zhou Y., Cao J., Yuan M., Lv H. (2021). Phosphorous doped g-C_3_N_4_ supported cobalt phthalocyanine: An efficient photocatalyst for reduction of CO_2_ under visible-light irradiation. J. Colloid Interface Sci..

[23] Khan I., Khan S., Wu S.-Y., Chen H.-T., Zada A., Linlin L., Ismail A., Ali S., Raziq F., Haider M. (2023). Synergistic Functionality of Dopants and Defects in Co-Phthalocyanine/B-CN Z-Scheme Photocatalysts for Promoting Photocatalytic CO_2_ Reduction Reactions. Small.

[24] Mo Y., Wang C., Xiao L., Chen W., Lu W. (2021). Artificial light-harvesting 2D photosynthetic systems with iron phthalocyanine/graphitic carbon nitride composites for highly efficient CO_2_ reduction. Catal. Sci. Technol..

[25] Ji Y.C., Yang R.Q., Wang L.W., Song G.X., Wang A.Z., Lv Y.W., Gao M.M., Zhang J., Yu X. (2020). Visible light active and noble metal free Nb_4_N_5_/TiO_2_ nanobelt surface heterostructure for plasmonic enhanced solar water splitting. Chem. Eng. J..

[26] Deng A., Zhao E., Li Q., Sun Y., Liu Y., Yang S., He H., Xu Y., Zhao W., Song H. (2023). Atomic Cobalt–Silver Dual-Metal Sites Confined on Carbon Nitride with Synergistic Ag Nanoparticles for Enhanced CO_2_ Photoreduction. ACS Nano.

[27] Chen L., Li H., Li H., Li H., Qi W., Zhang Q., Zhu J., Zhao P., Yang S. (2022). Accelerating photogenerated charge kinetics via the g-C_3_N_4_ Schottky junction for enhanced visible-light-driven CO_2_ reduction. Appl. Catal. B Environ..

[28] Zhang Q., Liu J., Wang D., Jin C., Fang L., Li X., Jiang Y., Wang X., Tian C. (2025). Coordination-induced in situ confinement of small-sized Ag nanoparticles on ultrathin C_3_N_4_ with strong metal–support interaction for enhanced selective CO_2_ photoreduction. Inorg. Chem. Front..

[29] Hu S., Qiao P., Yi X., Lei Y., Hu H., Ye J., Wang D. (2023). Selective Photocatalytic Reduction of CO_2_ to CO Mediated by Silver Single Atoms Anchored on Tubular Carbon Nitride. Angew. Chem. Int. Ed..

[30] Li S.Y., Zhang M., Qu Z.H., Cui X., Liu Z.Y., Piao C.C., Li S.G., Wang J., Song Y.T. (2020). Fabrication of highly active Z-scheme Ag/g-C_3_N_4_-Ag-Ag_3_PO_4_ (110) photocatalyst photocatalyst for visible light photocatalytic degradation of levofloxacin with simultaneous hydrogen production. Chem. Eng. J..

[31] Ong W.J., Tan L.L., Ng Y.H., Yong S.T., Chai S.P. (2016). Graphitic Carbon Nitride (g-C_3_N_4_)-Based Photocatalysts for Artificial Photosynthesis and Environmental Remediation: Are We a Step Closer To Achieving Sustainability?. Chem. Rev..

[32] Liu Y., Li J.M., Cao X.R., Wei X., Cao J.Y., Lin K.X., Zhou Y.Y., Ai Y.J. (2023). Hydrogenated Si-doped g-C_3_N_4_: Promising electrocatalyst for CO_2_ capture and conversion. Appl. Surf. Sci..

[33] Zhang L., Mao F.X., Zheng L.R., Wang H.F., Yang X.H., Yang H.G. (2018). Tuning Metal Catalyst with Metal-C_3_N_4_ Interaction for Efficient CO_2_ Electroreduction. ACS Catal..

[34] Omr H.A.E., Putikam R., Hussien M.K., Sabbah A., Lin T.Y., Chen K.H., Wu H.L., Feng S.P., Lin M.C., Lee H. (2023). Design of sculptured SnS/g-C_3_N_4_ photocatalytic nanostructure for highly efficient and selective CO_2_ conversion to methane. Appl. Catal. B-Environ. Energy.

[35] Dai Y.X., Wang Y.T., Zuo G.C., Kong J.J., Guo Y., Sun C., Xian Q.M. (2022). Photocatalytic degradation mechanism of phenanthrene over visible light driven plasmonic Ag/Ag_3_PO_4_/g-C_3_N_4_ heterojunction nanocomposite. Chemosphere.

[36] Zhuang C.Q., Chang Y., Li W.M., Li S.J., Xu P., Zhang H., Zhang Y.H., Zhang C., Gao J.F., Chen G. (2024). Light-Induced Variation of Lithium Coordination Environment in g-C_3_N_4_ Nanosheet for Highly Efficient Oxygen Reduction Reactions. ACS Nano.

[37] Sun L.L., Feng Y.B., Ma K., Jiang X.H., Gao Z.Y., Wang J.G., Jiang N., Liu X.S. (2022). Synergistic effect of single-atom Ag and hierarchical tremella-like g-C_3_N_4_: Electronic structure regulation and multi-channel carriers transport for boosting photocatalytic performance. Appl. Catal. B-Environ. Energy.

[38] Liu S., Zhou Q., Wen D., Wu C., Pan Y., Liu X., Huang Z., Li N. (2024). Particle Size-Dependent Charge Transfer Dynamics for Boosting CO_2_ Photoreduction over Ag/TiO2 Heterojunction. ACS Catal..

[39] Zhang S., Mo Z.Z., Wang J., Liu H.L., Liu P., Hu D., Tan T.X., Wang C. (2021). Ultra-stable oxygen species in Ag nanoparticles anchored on g-C_3_N_4_ for enhanced electrochemical reduction of CO_2_. Electrochim. Acta.

[40] Xu Q.L., Zhang L.Y., Cheng B., Fan J.J., Yu J.G. (2020). S-Scheme Heterojunction Photocatalyst. Chem.

[41] Lu C.H., Zhang P., Jiang S.J., Wu X., Song S.Q., Zhu M.S., Lou Z.Z., Li Z., Liu F., Liu Y.H. (2017). Photocatalytic reduction elimination of UO22+ pollutant under visible light with metal-free sulfur doped g-C_3_N_4_ photocatalyst. Appl. Catal. B-Environ..

[42] Ghafoor S., Inayat A., Aftab F., Duran H., Kirchhoff K., Waseem S., Arshad S.N. (2019). TiO2 nanofibers embedded with g-C_3_N_4_ nanosheets and decorated with Ag nanoparticles as Z-scheme photocatalysts for environmental remediation. J. Environ. Chem. Eng..

[43] Liu Y., Zhao Y., Li M., Liu Y. (2023). Annealing temperature effects on monolayer WS(2)-veiled Ag nanoparticle array for surface catalytic reaction. Spectrochim. Acta A Mol. Biomol. Spectrosc..

[44] Tafreshi S.S., Moshfegh A.Z., de Leeuw N.H. (2019). Mechanism of Photocatalytic Reduction of CO_2_ by Ag_3_PO_4_(111)/g-C_3_N_4_ Nanocomposite: A First-Principles Study. J. Phys. Chem. C.

[45] Shen R.C., Zhang L.P., Chen X.Z., Jaroniec M., Li N., Li X. (2020). Integrating 2D/2D CdS/α-Fe2O3 ultrathin bilayer Z-scheme heterojunction with metallic β-NiS nanosheet-based ohmic junction for efficient photocatalytic H2 evolution. Appl. Catal. B-Environ..

[46] Lin Y., Yang C.P., Wu S.H., Li X., Chen Y.J., Yang W.L. (2020). Construction of Built-In Electric Field within Silver Phosphate Photocatalyst for Enhanced Removal of Recalcitrant Organic Pollutants. Adv. Funct. Mater..

[47] Zhou B., Xu S., Wu L.Q., Li M.J., Chong Y.A., Qiu Y.C., Chen G.X., Zhao Y., Feng C.H., Ye D.Q. (2023). Strain-Engineering of Mesoporous Cs3Bi2Br9/BiVO4 S-Scheme Heterojunction for Efficient CO_2_ Photoreduction. Small.

[48] Zheng Z., Du T., Chen P., Yue Q., Wang H., Zhou L., Wang Y. (2024). 2D/1D nested hollow porous ZnIn_2_S_4_/g-C_3_N_4_ heterojunction based on morphology modulation for photocatalytic CO_2_ reduction. J. Environ. Chem. Eng..

[49] Zhang S.W., Feng H.N., Li C.Y., Cao X.D., Li H., Wu Y. (2024). The reduction mechanism of C1 product from carbon dioxide catalyzed by Ni-doped g-C_3_N_4_. Mol. Catal..

[50] Kawai K., Narushima T., Kaneko K., Kawakami H., Matsumoto M., Hyono A., Nishihara H., Yonezawa T. (2012). Synthesis and antibacterial properties of water-dispersible silver nanoparticles stabilized by metal-carbon σ-bonds. Appl. Surf. Sci..

[51] Posada-Pérez S., Vidal-López A., Solá M., Poater A. (2023). 2D carbon nitride as a support with single Cu, Ag, and Au atoms for carbon dioxide reduction reaction. Phys. Chem. Chem. Phys..

